# Rebuttal to: Neuroanatomical changes associated with age-related hearing loss and listening effort

**DOI:** 10.1007/s00429-021-02263-2

**Published:** 2021-04-12

**Authors:** Stephanie Rosemann, Christiane Thiel

**Affiliations:** 1 Biological Psychology, Department of Psychology, Department for Medicine and Health Sciences, Carl-von-Ossietzky Universität Oldenburg, Ammerländer Heerstraße 114-118, 26111 Oldenburg, Germany; 2 Cluster of Excellence “Hearing4all”, Carl von Ossietzky Universität Oldenburg, Ammerländer Heerstraße 114-118, 26111 Oldenburg, Germany

Dear Editor,

We have read the letter by Xiaoling Yin and Yun Zheng in critique to our research that was published as “Neuroanatomical changes associated with age-related hearing loss and listening effort” (Rosemann and Thiel 2020). We appreciate the concerns of the authors of this letter and hereby take the opportunity to respond to those comments.

The first issue that is raised in the letter is that it is complicated to exclude other risk factors in our study that may have led to an increase of hearing thresholds. We gathered data about health co-morbidities such as hypertension, diabetes and cigarette smoking in our sample which was not included in the paper. Please find this information presented in Table 1. Frequencies of risk factors across both groups did not significantly differ (all *p* > 0.1). Furthermore, we excluded participants that reported hearing loss resulting from ototoxic medications and or noise exposure.

**Table 1:** Health co-morbidities such as hypertension, diabetes and cigarette smoking across hard of hearing and normal-hearing participants

	Diabetes	Smoking	Hypertension
Hard of hearing (*n* = 38)	*n* = 1	*n* = 7	*n* = 9
Normal hearing (*n* = 33)	*n* = 0	*n* = 7	*n* = 7

Second, the letter states that the dependent variable that was used in our regression analysis was ranked listening effort rating. We would like to clarify that the dependent variable in this regression analysis was grey matter volume (or cortical thickness) and the independent variable was (the mean) listening effort. We would also like to add some information about the listening effort questionnaire: it consists of 17 listening situations in everyday life of different difficulty (e.g. seeing the speaker or not). For every situation, participants are asked to rate the listening effort they experience in that situation, for instance ‘How effortful is it to follow the conversation?’. All rating scales were 11-point Likert scales between 0 (not effortful at all) and 10 (extremely effortful). The mean rating value across all 17 situations was used as a marker of listening effort and entered in the linear regression analysis. The mean instead of the median was used here based on the assumption that the response options are equidistant. Using the (parametric) mean for Likert scales is also widely reported and considered appropriate in the literature (Carifio and Perla 2008; Harpe 2015; Norman 2010). Hence, the obtained mean listening effort across 17 questions can be regarded as a continuous interval variable and was used as independent variable in the regression.

We hope that the information above clarifies our findings.
